# Machine Learning for Mortality Analysis in Patients with COVID-19

**DOI:** 10.3390/ijerph17228386

**Published:** 2020-11-12

**Authors:** Manuel Sánchez-Montañés, Pablo Rodríguez-Belenguer, Antonio J. Serrano-López, Emilio Soria-Olivas, Yasser Alakhdar-Mohmara

**Affiliations:** 1 Escuela Politécnica Superior, Universidad Autónoma de Madrid, 28049 Madrid, Spain; manuel.smontanes@uam.es; 2 IDAL, Intelligent Data Analysis Laboratory, ETSE, Universitat de Valencia, 46100 Burjassot, Spain; parodbe@gmail.com (P.R.-B.); emilio.soria@uv.es (E.S.-O.); 3 Department of Physiotherapy, Universitat de Valencia, 46010 Valencia, Spain; yasser.alakhdar@uv.es

**Keywords:** COVID-19, survival analysis, machine learning, feature importance, graphical models

## Abstract

This paper analyzes a sample of patients hospitalized with COVID-19 in the region of Madrid (Spain). Survival analysis, logistic regression, and machine learning techniques (both supervised and unsupervised) are applied to carry out the analysis where the endpoint variable is the reason for hospital discharge (home or deceased). The different methods applied show the importance of variables such as age, O_2_ saturation at Emergency Rooms (ER), and whether the patient comes from a nursing home. In addition, biclustering is used to globally analyze the patient-drug dataset, extracting segments of patients. We highlight the validity of the classifiers developed to predict the mortality, reaching an appreciable accuracy. Finally, interpretable decision rules for estimating the risk of mortality of patients can be obtained from the decision tree, which can be crucial in the prioritization of medical care and resources.

## 1. Introduction

The COVID-19 pandemic is a great challenge for humanity, with more than 25 million confirmed cases as of 31 August 2020 according to the World Health Organization (WHO) [1]. The first clusters of COVID-19 cases were reported in December 2019 and January 2020. A search, on the same date, on clinicaltrials.gov for studies targeting the COVID condition showed more than 3100 registered studies [2,3]. This disease has affected the whole world with a large number of studies and review articles appearing about different aspects of this disease: possible symptoms and treatments [4,5,6,7,8,9], technological tools to combat the virus [10,11,12,13], epidemiological models of virus transmission [14,15,16], the detection of fake news related to COVID-19 [17,18], etc. This huge, ever-growing amount of work reflects the need to gather knowledge about this virus in all possible ways.

On the other hand, recent studies have shown that machine/deep learning techniques are technologies used in all branches of health sciences as elements of clinical decision support and as generators of new clinical knowledge [19,20,21]. In this regard, multiple research projects have been published using machine/deep learning techniques for the early detection of the COVID-19 virus [22,23,24,25], models applied to patients admitted to ICUs, which have been the clinical units most affected by the virus [26,27,28], and machine/deep learning applied in “omic” technologies to predict complications of COVID-19 [29,30]. The number of published papers about COVID-19 is continuously growing [31,32,33].

In this paper, we analyze the anonymized dataset obtained from the HMhospitals’ network in Madrid (Spain), thanks to their project COVID DATA SAVE LIVES [34]. In [35], several hypothesis contrasts and the chi-squared and Wilcoxon tests were performed using this dataset. However, model-based statistical techniques and machine learning techniques were uncovered. In this paper, we analyze the dataset using other statistical models (logistic regression and survival analysis), as well as supervised (decision trees, random forest, and Bayesian networks) and unsupervised (biclustering) machine learning techniques [36,37,38]. Therefore, a relevant contribution of this manuscript is the use of methods that allow direct extraction of clinical knowledge from these models.

From the application of the different methods, the great importance of the following variables is shown for the favorable evolution of the disease: age and gender of the patient, O_2_ saturation index, and place of origin. In addition, the unsupervised analysis makes it possible to establish the efficacy of some of the drugs used. Finally, the validity of the classifiers developed to predict mortality is checked where the outcome variable is hospital discharge (the patients were reported as home or deceased), with the best model reaching a value of 0.89 in AUC for the test set (the worst model reaching a value of 0.77). Interpretable decision rules for estimating the risk of mortality of the patient can be obtained from the decision trees, which can be crucial in the prioritization of medical care and resources.

This paper has the following structure: Section 2 describes the data and the methods applied to these data; Section 3 applies different statistical models (survival analysis and logistic regression), as well as machine learning methods; finally, Section 5 contains the conclusions obtained from the analysis of the results.

## 2. Data and Methods

This section outlines the data analyzed, as well as the methods used. Given the large number of methods used, these methods are briefly described.

### 2.1. Data

The anonymized dataset was obtained from the HM hospitals’ HERsystem, which was openly released on April 25th, thanks to its project COVID DATA SAVE LIVES. The research groups that wants to analyze it should present a project beforehand, and said project is to be approved by the corresponding board of experts [34].

The dataset contains several CSV files. The first one contains 2307 patients and 29 variables, is focused on data on the admission of the patient, and has the following fields:

- Patient ID
- Age and gender
- COVID diagnostic (confirmed/pending confirmation)
- ER date in
- ER specialty, ER diagnostic, and destination after ER
- First and last constant measurements in the ER (heart rate, temperature, minimum and maximum arterial pressure, O_2_ saturation in blood)
- Admission date to the hospital
- ICU date in, ICU date out, and number of days in the ICU (if applicable)
- Discharge date and destination (home/deceased/transferred to other hospital/voluntary discharge/transferred to a socio-sanitary center)

This dataset was expanded with another variable, Residential_Institution, which indicates whether the patient comes from a nursing home. This information can be extracted from another CSV files provided by HM hospitals.

For the preprocessing tasks of the first dataset, we considered only patients with confirmed COVID-19 that were admitted to the hospital after the ER and with a discharge destination equal to “home” or “deceased”. On the other hand, variables with more than 30% missing values were removed, and the ER diagnostic (45 categories) was simplified to one of the following categories: difficulty breathing, catarrhal picture, cough, fever, oncological patient deterioration, and other.

The cleaned dataset contained 25 variables and 1696 confirmed COVID-19 patients with 61% male and 39% female patients, with a mean of age of 66.5 years (the youngest/oldest patients were 0/106 years old, respectively). Common continuous variables caused by SARS-CoV-2, such as oxygen saturation at admission, were collected for 82.6% of patients, with 18% of patients having less than 90% oxygen saturation, which are not clinically bad results. Temperature was available for 80.5% of the data collected, with 10% of patients presenting a temperature > 38.0 °C (one patient had a temperature of 40.1 °C, the highest reading in the dataset). The patients had a mean of diastolic and systolic blood pressures of 130–70 mmHg. Most of the patients had only one measurement for each constant. Therefore, we only took into account the first measurement. Finally, sixteen-point-six-three percent of patients from this dataset were deceased, with the last update received on April 25th.

The other CSV file we used contained the list of drugs and doses administered by medical personnel to each patient.

### 2.2. Data Analysis Methods

#### 2.2.1. Survival Data Analysis

Survival analysis is a method for investigating the time elapsed until an event occurs, in our case the death of the patient. There are different methods to perform this analysis, and here, we chose the simplest and most common method, the Kaplan–Meier method—a non-parametric method used to estimate the survival probability from observed survival times [39,40].

The survival probability at time $t_{j}$, $P(t_{j})$, is calculated as follows in Equation (1),

(1) $$P\left(t_{j}\right)=P\left(t_{j-1}\right)\left(1-\frac{d_{j}}{n_{j}}\right)$$

where $n_{j}$ is the number of patients alive just before $t_{j}$ and $d_{j}$ is the number of events (deaths) at $t_{j}$. The probability of survival can then be represented by the Kaplan–Meier curve, which provides a very useful visualization of the evolution of mortality in a given disease.

#### 2.2.2. Logistic Regression

Logistic regression belongs to the group of models known as generalized linear models. In this type of model, there are two operations: first, a linear operation where a multiple linear regression is obtained from the independent or predictor variables; second, a sigmoid function is applied to estimate the probability of belonging to a given class (Equation (2) [41]):

(2) $$P\left(class|x_{1},\cdots ,x_{N}\right)=\frac{1}{1+e^{-(w_{0}+\sum_{k=1}^{N}w_{k}\cdot x_{k})}}$$

Here, $w_{k}$ (k∈[0, N]) are the model parameters, *N* is the number of predictor variables, and $x_{k}$ are these variables for a given patient. Logistic regression is the classic statistical model used in classification problems [37].

#### 2.2.3. Bayesian Network

A Bayesian Network (BN) is a probabilistic graphical model composed of two different parts: first, the graphical structure (directed acyclic graph) that defines the relationship between variables and, second, the probabilities established between these variables [42]. The elements of a Bayesian network are as follows [38]:

- A set of variables (continuous or discrete) forming the network nodes.
- A set of directed links that connect a pair of nodes. If there is a relationship with direction X→Y, it is said that X is the parent of Y.
- Each node $X_{i}$ is associated with a conditional probability function $P(X_{i}|Parents\left(X_{i}\right))$ that takes as the input a particular set of values for the node’s parent variables and gives the probability of the variable represented by the node $X_{i}$.
- The graph has no directed cycles.

The knowledge is reflected by the relationships established in the graph nodes and gives the conditional probability values of the variables represented in each node. Those probabilities are estimated using the dataset.

In this paper, we applied an evolution of the basic model of naive Bayes): TAN (Tree Augmented Network) [42]. In naive Bayes, the hypothesis is to assume that the predictive variables are conditionally independent given the outcome variable. The conditional probability $P\left(x_{1},\cdots ,x_{N}|class\right)$ is factorized as $\prod_{i=1}^{N}P\left(x_{i}|class\right)$ [42]. This factorization simplifies the calculation and analysis of the conditional probability from experimental data. In addition, it also simplifies the inference process from new data. On the other hand, TAN is an extension of naive Bayes in which each variable is allowed to have another parent outside the class node. The idea is to build a Bayesian network tree for all predictive variables and complete the model with naive Bayes. The TAN algorithm forms a tree with the predictive variables and then adds edges to the class node [42].

#### 2.2.4. Decision Tree

A decision tree is a hierarchical model for supervised learning in which the decision tree’s local region is identified in a sequence of recursive divisions as a number of steps; it is composed of internal decision nodes and terminal leaves [37]. Decision trees have two main advantages and therefore have been used in this work [36]:

- A decision tree is a non-parametric model; it does not assume any parametric form for class densities, and the structure of the tree is not fixed a priori. Rather, the tree grows during learning depending on the complexity of the problem.
- They are explanatory models as opposed to other more powerful models, such as neural networks, in which extracting knowledge from them is extremely complex.

#### 2.2.5. Random Forest

Random forest is a substantial modification of bagging that builds up a large collection of decorrelated trees and then averages them [43]. In many problems, the performance of random forests is very similar to boosting, and they are easier to train and tune. Furthermore, Fernandez-Delgado et alt demonstrated its superiority in a comparison of several algorithms on different problems, so this algorithm can be considered as a reference [44]. As a result, random forests are popular and are implemented in a variety of packages. Another important reason for using random forests is that they also provide an analysis of the importance of each variable in order to solve a certain problem [45] (in our case, predicting exitus).

#### 2.2.6. Biclustering

A classic use of unsupervised learning is clustering algorithms, whose mission is to obtain areas of high data density [36,37]. Once these areas are obtained, clustering provides information about the variables/patterns that characterize them, which can be easily interpreted, allowing us to extract knowledge from the data.

In this work, we applied biclustering [46], which is a particular case of clustering that, in our opinion, provides additional information when compared to other clustering methods such as k-means [37]. A clustering algorithm such as k-means tries to group the rows or the columns of the dataset, while a biclustering algorithm tries to group a certain set of rows and columns together. If we apply k-means to our problem, its objective would be to find groups of patients with similar characteristics. However, if we apply biclustering techniques, the objective is much more ambitious: to find, together, groups of patients and groups of variables within the population. This is much more powerful because it allows a better segmentation of the population. This fact allows extracting more knowledge of the problem under analysis.

### 2.3. Software

We used the R and Python programming languages for developing the scripts to create and analyze the models. In Python, we extensively used the standard libraries Sklearn [47], Pandas [48], Numpy [49], and Matplotlib [50]. For R, we mainly used the following libraries: Caret [51], e1701 [52], and ggplot2 [53].

## 3. Results

### 3.1. Survival Analysis

The dataset was collected over a time interval of 78 days. The records began with the first admission to the emergency department on 5 February 2020 and continued until the last discharge in 23 April 2020.

**Figure 1:**
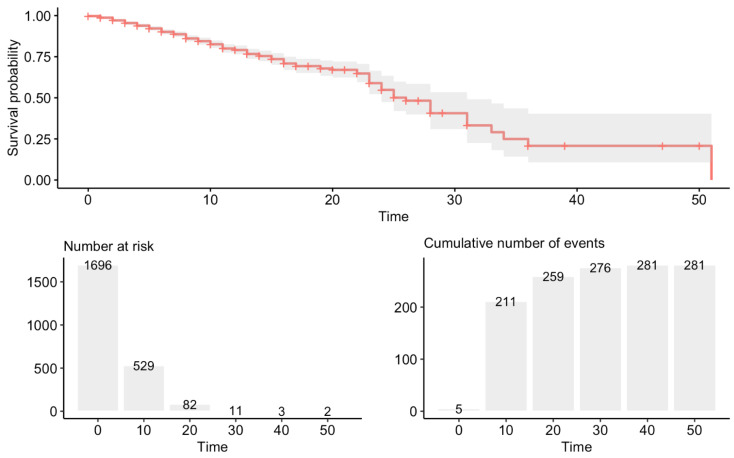
Survival probability over time.

In 99% of the temporary stays, the number of deaths was lower than the number of patients discharged and sent home. In stays of less than 20 days, the highest percentage of deaths (42%) coincided with the records of zero days of duration. In this dataset, the average time at the hospital was 27.09 ± 1.56 days (Figure 1).

Only four very significant variables were found (log-rank test): age, residential institution, O_2_ saturation, and heart rate. Furthermore, the simplified diagnosis showed statistical significance (Table 1).

**Figure 2:**
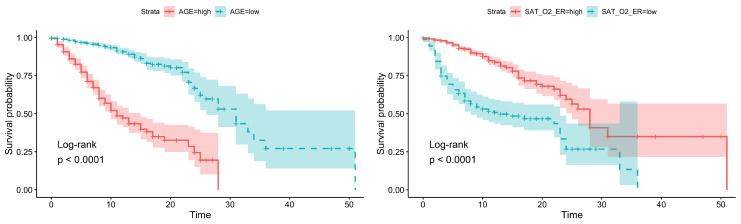
Survival analysis: (**Left**) Age. (**Right**) O_2_ saturation.

**Figure 3:**
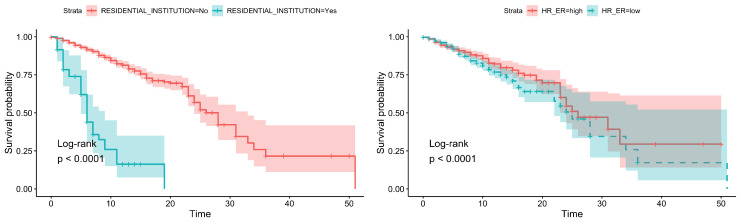
Survival analysis: (**Left**) Residential institution. (**Right**) heart rate.

The dependence of mortality on these variables is clear when Kaplan–Meier curves are represented; see Figure 2 and Figure 3. It can be clearly seen that there is a strong dependence on age and residential institution (the curves are more separated).

### 3.2. Supervised Learning Analysis

All the developed models in this section were constructed and tested using the same preprocessed dataset. Categorical variables were replaced by numerical dummy variables. Then, the dataset was split into the training set (70% patients) and test set (30% patients) using stratified sampling according to the discharge destination statistics (“home”/“deceased”). These two sets are mutually exclusive. The training set was used for constructing the models, while the test set was used for estimating their generalization capability. Additionally, in the logistic regression model, the variables were standardized according to their training set statistics. Once a model was constructed, its optimal classification threshold was computed as the threshold corresponding to the ROC point in training closest to the (FPR = 0, TPR = 1) point.

In order to evaluate the reliability of the supervised learning models, a 10-fold resampling technique was applied to the training set. This allows estimating a confidence interval for the performance of each model. Finally, we checked that the performance for the test set was contained in this confidence interval, confirming the reliability of our results.

#### 3.2.1. Logistic Regression

**Figure 4:**
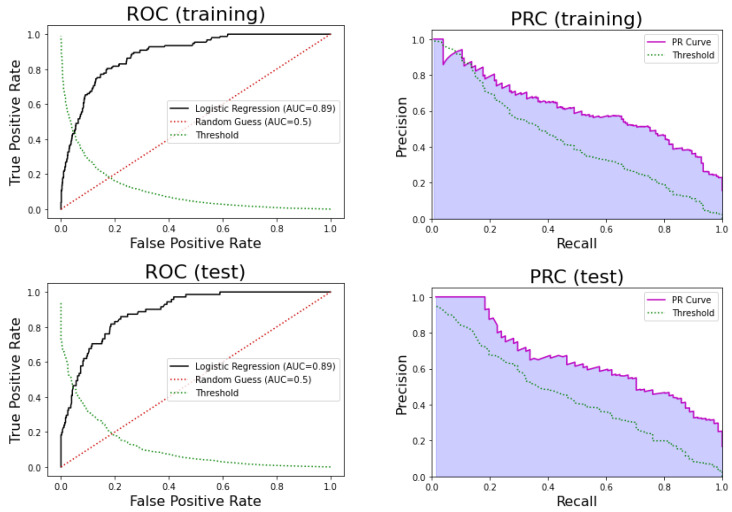
(**Top**) Receiver Operating Curve (ROC) of the logistic regression model in training (**left**) and testing (**right**). (**Bottom**) Precision-Recall Curve (PRC) in training (**left**) and testing (**right**).

Figure 4 shows the Receiver Operating Curve (ROC) and Precision-Recall Curves (PRCs) for the logistic regression. We can see that the performance of the model for the training set was very similar to that observed for the test set. On the one hand, logistic regression obtained an AUC of 0.89, sensitivity = 0.80, specificity = 0.83, PPV = 0.46, and NPV = 0.96 for the training set. On the other hand, for the test set, the model obtained an AUC of 0.89, sensitivity = 0.82, specificity = 0.81, PPV = 0.47, and NPV = 0.96. The 95% confidence interval for the AUC estimated using the k-fold in training was [0.86, 0.91]. Therefore, the AUC for the test set was contained in that interval, and there was no significant difference between the k-fold sample mean and the test AUC (*p*-value = 0.81 one sample *t*-test). We can conclude that this model is highly reliable.

The coefficients, standard error, and *p*-values for the significance of the variables are shown in Table 2.

The most important variables for the logistic regression were then age, O_2_ saturation, residential institution, and oncological patient deterioration.

#### 3.2.2. Decision Tree

**Figure 5:**
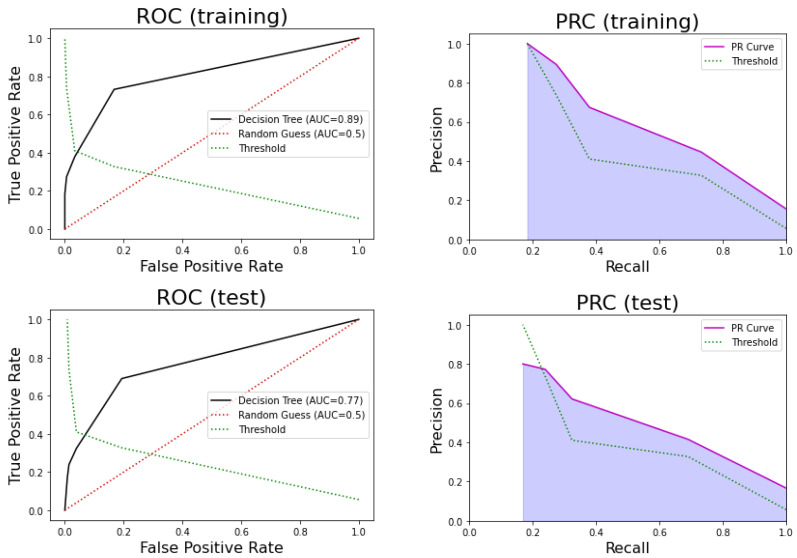
(**Top**) Receiver Operating Curve (ROC) for the decision tree model in training (**left**) and testing (**right**). (**Bottom**) Precision-Recall Curve (PRC) in training (**left**) and testing (**right**).

One of the problems of decision trees is that they can easily overfit the training dataset (the test set performance is much poorer than the training set performance). The reason is that these models tend to learn too specific rules that are not statistically significant. In order to prevent this, we used 10-fold CV for the training set, resulting in a value of five leaf nodes. The number of leaf nodes determines the number of rules in the tree. Figure 5 shows the ROC and precision-recall curves for this model.

**Figure 6:**
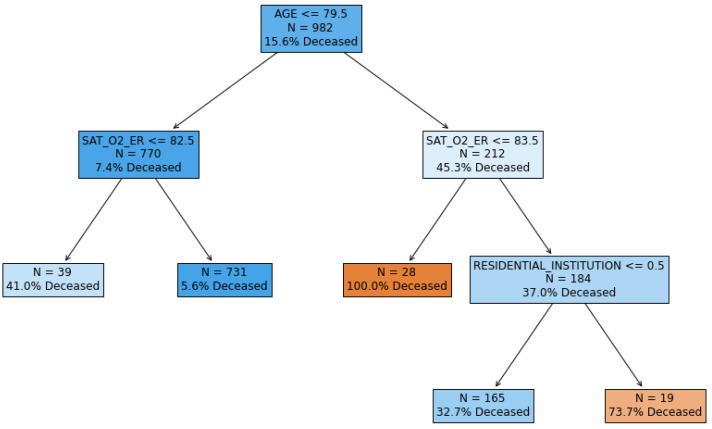
Decision tree.

In Figure 6, we can observe the constructed decision tree.

For the training set, this model obtained an AUC = 0.81, sensitivity = 0.73, specificity = 0.83, PPV = 0.45, and NPV = 0.94. For the test set, the model obtained an AUC = 0.77, sensitivity = 0.69, specificity = 0.81, PPV = 0.42, and NPV = 0.93. Therefore, we concluded that the performance in terms of these statistics was not very good. On the other hand, the 95% confidence interval for AUC estimated using the k-fold in training was [0.75, 0.81]. Thus, the AUC for the test set was contained in that interval, and there was no significant difference between the k-fold sample mean and the test AUC (*p*-value = 0.43 one sample *t*-test).

However, one of the main advantages of decision trees is that they can be very easily interpreted, as can be seen in Figure 6. Moreover, they can be rewritten as an equivalent set of rules. In Table 3, we list the set of rules equivalent to the constructed decision tree. The support (percentage of patients that satisfy the rule) and exitus probability in patients satisfying the rule were also computed. Since the rules are mutually exclusive (that is, any patient satisfies one and only one rule), they can be considered as a way to segment patients. That is, the decision tree partitions the patients into five different segments according to their probability of exitus, and each segment can be described with a decision rule.

In Table 3, we can observe that the statistics of the five segments in the training set are very similar to the statistics for the test set, confirming the robustness of this approach.

#### 3.2.3. Random Forests

**Figure 7:**
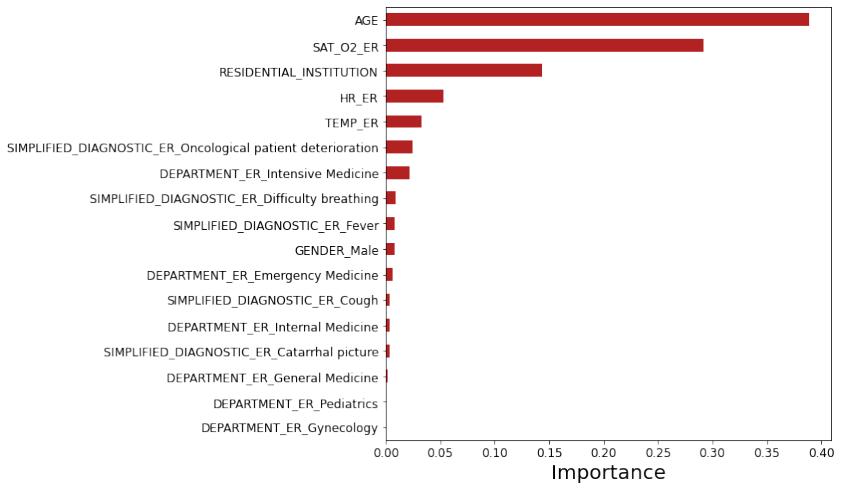
Importance of the variables according to the random forest model.

This model was trained using 501 estimators and a maximum depth of three levels using the same methodology as decision trees. One of the outputs of the model is the importance of each variable according to the tree statistics of the training set (see Figure 7). We can observe that the five most predictive variables of patient’s exitus were age, O_2_ saturation, residential institution, heart rate, and temperature, all measured in the Emergency Room (ER).

**Figure 8:**
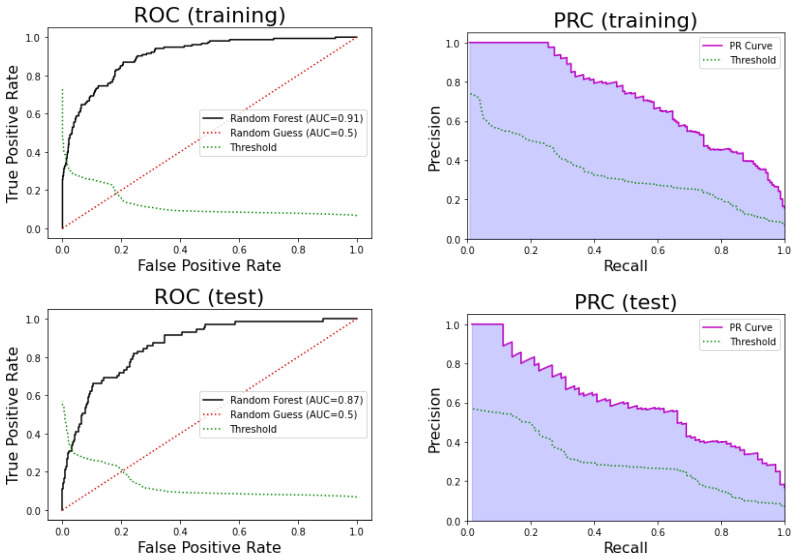
(**Top**) Receiver Operating Curve (ROC) of the random forest model in training (**left**) and testing (**right**). (**Bottom**) Precision-Recall Curve (PRC) in training (**left**) and testing (**right**).

For the training set, this model obtained an AUC = 0.90, sensitivity = 0.87, specificity = 0.79, PPV = 0.44, and NPV = 0.97, while for the test set, the model obtained an AUC = 0.87, sensitivity = 0.82, specificity = 0.75, PPV = 0.40, and NPV = 0.95. On the other hand, Figure 8 shows the ROC and precision-recall curves of this model for the training and test sets. We can observe that the performance of the model is good, but it degrades slightly for the test set.

The 95% confidence interval for AUC estimated using the k-fold in the training is [0.86, 0.89]. Therefore, the AUC for the test set is contained in that interval, and there is no significant difference between the k-fold sample mean and the test AUC (*p*-value = 0.66 one sample *t*-test). We can also conclude that this model is highly reliable.

#### 3.2.4. Bayesian Models

The Bayesian model selected was the discrete tree augmented naive Bayes. Consequently, continuous variables were discretized into three levels of equal frequency. The set of significant variables (*p* < 0.05, $\chi^{2}$ test with the class) yielded the best results. This set is formed by discretized age, residential institution (yes/no), discretized O_2_ saturation, department (Emergency Medicine/General Medicine, Gynecology/Intensive Medicine, Internal Medicine/Pediatrics/Traumatology), and simplified diagnostic (catarrhal picture/cough/difficulty breathing/fever/oncological patient deterioration/Other).

The quality of the model fit to the data was measured using the likelihood of the model and the Bayesian correction of the probability. These metrics were obtained using the standard 10-fold cross-validation procedure.

**Figure 9:**
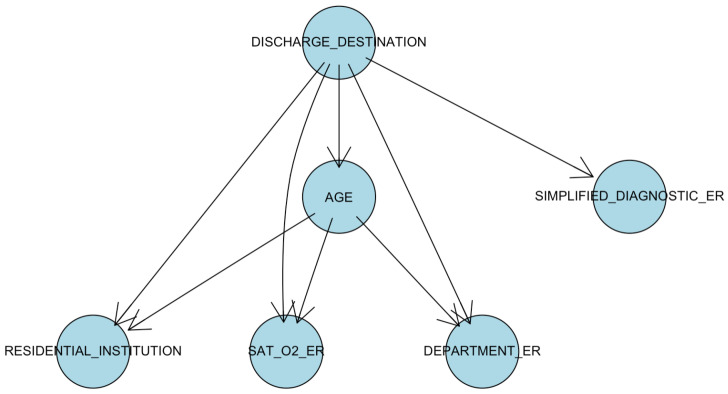
Tree augmented naive Bayes. The age was discretized considering as the cut-off values of 60 and 75 years and of 92% and 95% for O_2_ saturation.

We can observe in Figure 9 the graph obtained with the best AIC score (Akaike’s Information Criterion). The class variable (discharge destination, with possible values deceased/home) is related to all the significant variables. Furthermore, age modifies the influence of O_2_ saturation, residential institution, or ER department.

**Figure 10:**
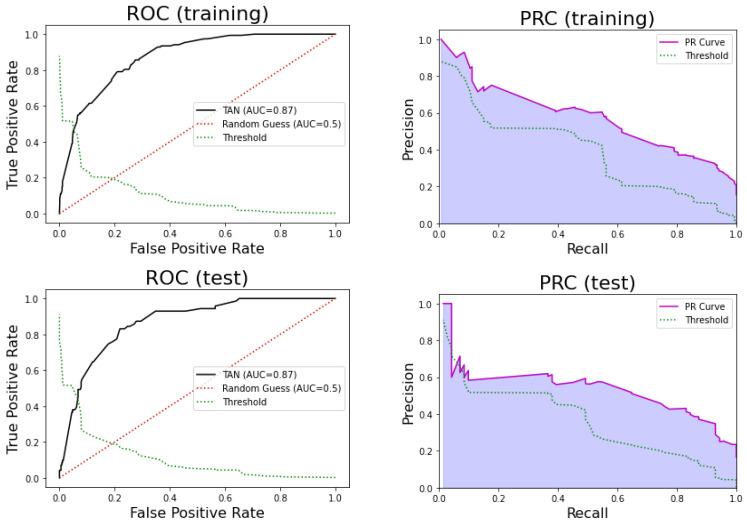
(**Top**) Receiver Operating Curve (ROC) for the TAN model in the training (**left**) and testing (**right**) sets. (**Bottom**) Precision-Recall Curve (PRC) in the training (**left**) and testing (**right**) sets.

For the training set, this model obtained an AUC of 0.87, sensitivity = 0.79, specificity = 0.79, PPV = 0.41, and NPV = 0.95. For the test set, the model obtained an AUC of 0.87, sensitivity = 0.77, specificity = 0.79, PPV = 0.43, and NPV = 0.95. Figure 10 shows the ROC and precision-recall curves for this model.

The 95% confidence interval for AUC estimated using the k-fold in training is [0.83, 0.89]. Therefore, the AUC for test set is contained in that interval, and there is no significant difference between the k-fold sample mean and the test AUC (*p*-value = 0.50 one sample *t*-test). We can also conclude that this model is highly reliable.

We conclude that the performance of this model is good, and it also provides an interesting description of the relationships of the significant variables with the class. Since age and O_2_ saturation are discretized into a small range of values, these relationships can be easily explored.

#### 3.2.5. Models’ Comparison

**Figure 11:**
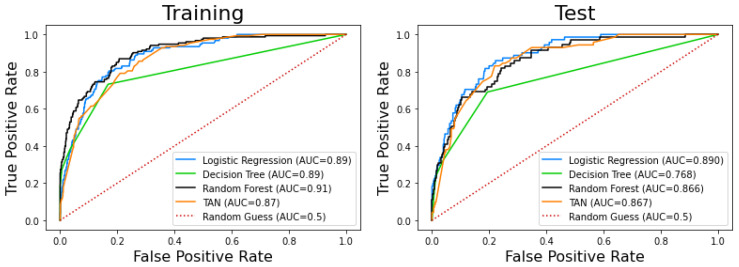
Receiver Operating Curves (ROCs) of the models. (**Left**) Training set. (**Right**) Testing set.

**Figure 12:**
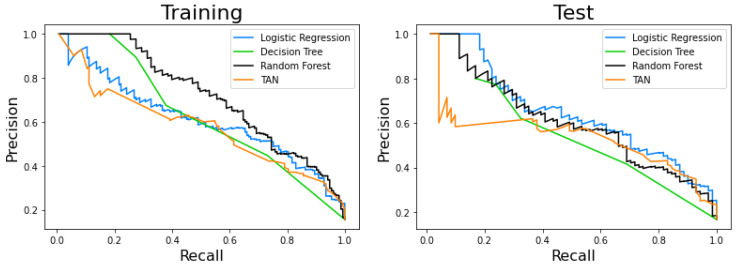
Precision-recall curve of the models. (**Left**) Training set. (**Right**) Testing set.

Table 4 and Table 5 summarize the results described above. Likewise, Figure 11 and Figure 12 jointly represent the ROCs and PCRs for the different models. It should be noted that all of them present good results in terms of AUC, which is one of the most used indexes in clinical classification problems.

Finally, we checked whether the models were statistically different; Table 6 shows the results of this comparison. We can observe that all pairs show significant differences except logistic regression-TAN and random forest-TAN. On the other hand, the decision tree is the most different model.

### 3.3. Unsupervised Learning Analysis

#### Biclustering

**Figure 13:**
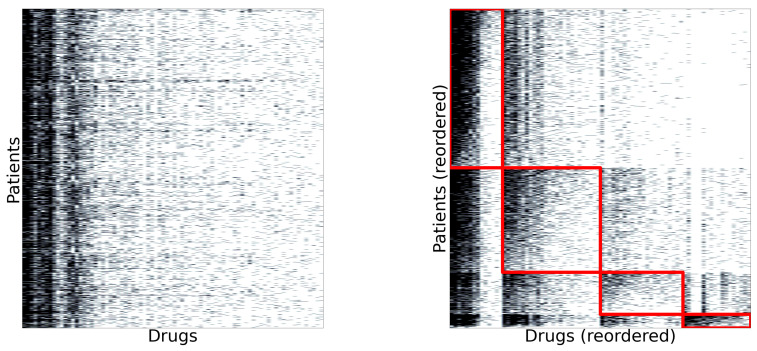
(**Left**) Original patient-drug matrix. (**Right**) Biclusters found by the co-clustering algorithm.

The patient-drug dataset was segmented using a biclustering algorithm. First, only patients presented in the first dataset were taken into account. On the other hand, only drugs administered to at least 50 patients were considered. Figure 13 (left) shows the original dataset. Each black point indicates that a particular drug (column) was administered to the patient (row). We can observe that some drugs were administered more frequently than others, but there was no apparent structure in the dataset. Figure 13 (right) shows the dataset reordered by the biclustering algorithm. It found four biclusters, each one marked by a red square.

**Figure 14:**
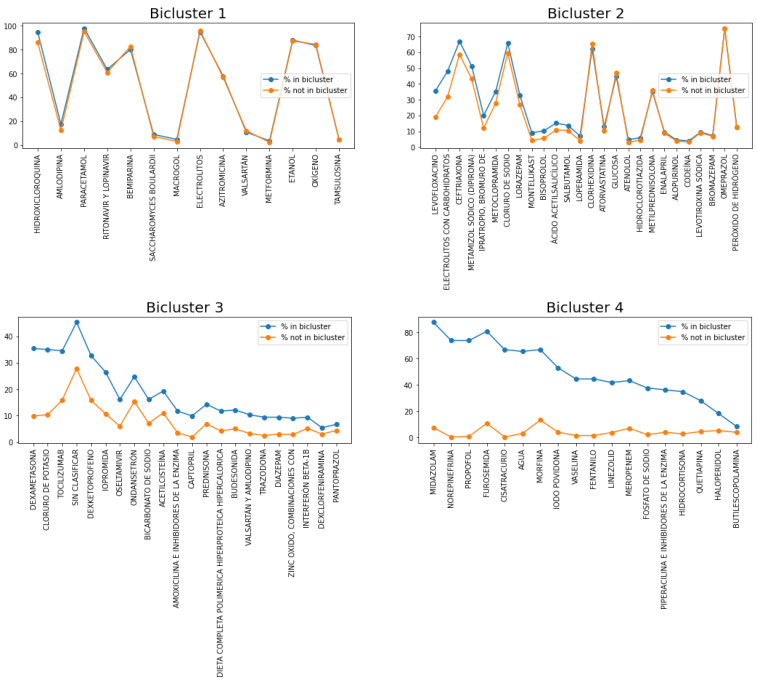
Representative drugs of each bicluster and their percentage of use for patients belonging to the bicluster versus the percentage for patients not in the bicluster.

Figure 14 shows the representative set of drugs in each bicluster. The blue line represents the % of patients in the bicluster where the drug was administered, while the orange line represents that frequency of patients not belonging to the bicluster. Therefore, the larger that difference, the more characteristic the drug is of that bicluster.

First, we can observe that the percentage of use of the representative drugs of Bicluster 1 is very similar to that percentage in the other clusters (Figure 14, top left). Representative drugs in this cluster are lopinavir/ritonavir (which are HIV protease inhibitors that, at the beginning of the COVID pandemic, were used in an experimental phase; however, the WHO stopped its trials for lack of efficacy [54]), hydroxychloroquine (an antimalarial drug that has also been proposed for its use against COVID), and oxygen management (it is worth remembering the need for mechanical ventilation at the beginning of the COVID pandemic), among others.

Likewise, Bicluster 2, the percentage of use of the representative drugs, is also very similar in the other clusters, except for levofloxacin, which is commonly used for urinary tract infections; therefore, the increase in the rate of mortality could be due to older patients and having a lower O_2_ saturation.

Regarding Bicluster 3, the high use of tocilizumab is interesting (Figure 14, bottom left), which is a humanized monoclonal antibody against the Interleukin-6 Receptor (IL-6R), which is used for Cytokine Release Syndrome (CRS). This is a systemic inflammatory response that can be triggered by a variety of factors, and its symptoms are fever, fatigue, and headache, among others. Furthermore, corticosteroids have also been used in a significant proportion. Oseltamivir has also been used in this bicluster, which is an antiretroviral commonly used for Influenzavirus A. Interestingly, there is a higher % of ondansetron in this bicluster, which is an antiemetic drug for nausea caused by chemotherapy, and therefore, it could be that the mortality rate was due to the high proportion of oncology patients in this bicluster. In fact, one of the significant variables in logistic regression was oncological patient deterioration. To conclude, the high proportion of anesthetic, analgesic, and sedative drugs in the last bicluster in comparison with the others is striking, which is why it could be called “the palliative bicluster”.

The above results were obtained by analyzing only the patient-drug dataset. We now use the first dataset analyzed in this paper (with variables such as age, discharge destination, etc.), and we calculate the statistics of these variables in each of the biclusters. Table 7 shows these statistics, and we can see a surprising result: the patients belonging to each bicluster show very different statistics from the others in terms of age, O_2_ saturation, % ICU, and % deceased. Note that these variables have not been used to obtain the biclusters. In Table 7, we observe that patients in Bicluster 1 were younger (62±15 years), presented a higher O_2_ saturation in the ER (94±4), and only 0.7% of them entered the ICU. The percentage of exitus in this bicluster is only 1.5%. Biclusters 2, 3, and 4 show increasing probabilities of entering the ICU, increasing probabilities of exitus, and increasing average ages. On the other hand, the mean O_2_ saturation decreases across these biclusters. We conclude that it can be clearly seen that each bicluster contains patients at different stages of the disease, from least to greatest severity.

## 4. Discussion

We analyzed a dataset of 1696 confirmed COVID-19 patients in the region of Madrid (Spain) that were hospitalized after visiting the emergency room. This dataset contains 61% of patients of the male gender. The percentage of exitus in this dataset is 16%. On the other hand, ninety-four percent of the deceased patients are older than 64 years, which clearly confirms that the COVID-19 pandemic is affecting the nucleus of the older population. In fact, the survival analysis reveals that age is a statistically significant predictor variable of decease, as well as residential institution, % O_2_ saturation, heart rate, and simplified diagnostic (see Table 1). These statistically significant variables agree with the importance of the variables analyzed with random forest (see Figure 7), where age is the most important predictor variable.

Logistic regression was the supervised machine learning model with the best predicting performance in the test set, reaching an AUC of 0.890, a sensitivity of 81.69%, and a specificity of 81.46% (see Table 4 and Figure 4). The most statistically significant variables in this model were age, gender, oxygen saturation, residential institution, and oncological patient deterioration.

On the other hand, decision trees allowed us to identify five segments of patients, each having clearly different mortality rates (Table 3). These segments are determined by the variables age, O_2_ saturation, and residential institution (see Figure 6) and show a very robust behavior in testing (very similar performance to that shown in training). These types of rules can be very useful in the ER to easily and quickly estimate a patient’s risk of dying. It is interesting that the variable RESIDENTIAL_INSTITUTION is associated with the segment with a higher risk of dying (71.4 % in testing). This indicates that patients in Madrid coming from a residential institution had a significantly higher risk of dying, which is compatible with the precarious position of Spanish residential institutions in relation with COVID-19 management [55].

In the Tree Augmented naive Bayes Network (TAN), the set of significant variables (see Figure 9) is formed by discretized age (two cut-offs: 60 and 75 years old), discretized O_2_ saturation (two cut-offs: 92 and 95%), residential institution (yes or no), ER department, and ER simplified diagnostic, which are the same variables that can be seen in Figure 7 (variables ordered by importance according to random forest). In fact, the TAN and random forest models have very similar AUC values.

Likewise, unsupervised machine learning models were used, specifically biclustering algorithms. We obtained four biclusters, each of them characterized by a particular set of drugs.

To conclude, this pandemic has forced health care workers to use any type of assistance. Given the absence of controlled clinical studies, it has been a time of change and adaptation. What seemed like a good option yesterday the next day has changed. For example, lopinavir/ritonavir began to be used, but in a news release on 4 July 2020 [54], WHO accepted the recommendation from the Solidarity Trial’s International Steering Committee to discontinue the trial’s lopinavir/ritonavir arm because it was producing little or no reduction in the mortality of hospitalized COVID-19 patients when compared to standard care. Furthermore, hydroxychloroquine treatment, an experimental antimalarial drug against COVID, was also used, and initially, its trials appeared to be encouraging; however, recently, on 1 July 2020, the FDA warned [56] of the risk of heart rhythm problems.

## 5. Conclusions

The world is facing a pandemic with profound health and socioeconomic implications. There is a generalized collapse of health systems, and projects such as COVID DATA SAVE LIVES are essential for artificial intelligence to provide healthcare staff with tools to speed up decision-making. This article attempts to bring knowledge to this disease. However, a study of these characteristics presents several problems: (a) the data do not come from a controlled study; (b) they have been collected in complex health situations; and (c) they are from a specific population that goes to these hospital centers. In spite of this, the usefulness of advanced data analysis was shown in order to extract knowledge.

This paper confirms that the elderly have a very high risk of dying from COVID-19. Supervised machine learning models show other variables that are important for predicting the evolution of the disease such as O_2_ saturation, ER department, and ER simplified diagnostic. Simple and interpretable decision rules for estimating the risk of mortality of the patient can be obtained from the decision trees, which can be crucial in the prioritization of medical care and resources.

Finally, we showed that unsupervised learning algorithms allow a global analysis of the set of drugs administered to the patient population. This allowed us to automatically identify groups of patients with very different evolutions. This allows analyzing the different therapeutic decisions and the impact on the evolution of the disease.

## Figures and Tables

**Table 1:** Significant variables in the survival analysis. The continuous variables were discretized using the maximally selected rank statistics to provide a value of a cutpoint that corresponds to the most significant relation with the outcome. Those variables with *p* < 0.05 are highlighted in bold.

Feature	Sig.	Optimal Cutpoint	Mean Time ± StdError
Age	**<0.0001**	79.0	high = 14.12 ± 0.70, low = 31.53 ± 1.95
Residential Institution	**<0.0001**		yes = 7.74 ± 0.91, no = 27.79 ± 1.61
Temperature	0.72	35.9	high = 27.88 ± 1.79, low = 20.09 ± 1.50
Heart Rate	**<0.0001**	89.0	high = 29.34 ± 2.48, low = 25.81 ± 2.21
O_2_ saturation	**<0.0001**	86.0	high = 30.44 ± 2.14, low = 16.65 ± 1.26
Gender	0.42		female = 31.99 ± 3.01, male = 25.36 ± 1.70
Department	0.36		Emergency Medicine 26.71 ± 1.59 General Medicine 40.48 ± 4.01 Gynecology 51.00 ± 0.00 Intensive Medicine 24.50 ± 9.03 Internal Medicine 30.76 ± 6.93 Pediatrics 51.00 ± 0.00 Traumatology 12.50 ± 1.06
Simplified Diagnostic	**0.019**		Catarrhal Picture 25.29 ± 1.83 Cough 30.22 ± 6.27 Difficulty Breathing 25.35 ± 2.26 Fever 39.92 ± 2.71 Oncological Patient Deterioration 23.34 ± 3.85 Other 27.02 ± 3.56

**Table 2:** Coefficients, standard error, and *p*-values for the significance of the variables in the logistic regression. Those variables with *p* < 0.05 are highlighted in bold.

Variables	Estimate	Std Error	*p*-Value
Age	1.738	0.174	**<0.001**
Gender	0.207	0.111	0.061
$O_{2}$ Saturation	0.102	1.277	**<0.001**
Residential Institution	0.082	1.329	**<0.001**
Oncological Patient Deterioration	0.078	1.277	**<0.001**

**Table 3:** Rules extracted from the decision tree.

Rule	Support (Training)	Deceased (Training)	Support (Test)	Deceased (Test)
(AGE<80) and $\left(SAT\;O_{2}<83\right)$	4.0% (N = 39)	41.0%	3.5% (N = 15)	40.0%
(AGE<80) and $\left(SAT\;O_{2}\geq 83\right)$	74.4% (N = 731)	5.6%	72.4% (N = 309)	7.1%
(AGE≥80) and $\left(SAT\;O_{2}<84\right)$	2.9% (N = 28)	100%	3.5% (N = 15)	80.0%
(AGE≥80) and $\left(SAT\;O_{2}\geq 84\right)$ and (notRESIDENTIAL_INSTITUTION)	16.8% (N = 165)	32.7%	19.0% (N = 81)	32.1%
(AGE≥80) and $\left(SAT\;O_{2}\geq 84\right)$ and (RESIDENTIAL_INSTITUTION)	1.9% (N = 19)	73.7%	1.6% (N = 7)	71.4%

**Table 4:** Performance of the supervised models on the training set. PPV: Positive Predictive Value. NPV: Negative Predictive Value.

Model	AUC	Sensitivity	Specificity	PPV	NPV
Logistic Regression	0.89	0.80	0.83	0.46	0.96
Decision Tree	0.81	0.73	0.83	0.45	0.94
Random Forest	0.90	0.87	0.79	0.44	0.97
Tree Augmented Naive Bayes	0.87	0.79	0.79	0.41	0.95

**Table 5:** Performance of the supervised models on the test set. PPV: Positive Predictive Value. NPV: Negative Predictive Value.

Model	AUC	Sensitivity	Specificity	PPV	NPV
Logistic Regression	0.89	0.82	0.81	0.47	0.96
Decision Tree	0.77	0.69	0.81	0.42	0.93
Random Forest	0.87	0.82	0.76	0.40	0.95
Tree Augmented Naive Bayes	0.87	0.77	0.79	0.43	0.95

**Table 6:** Pairwise comparison of the test ROC curves (Delong’s paired test).

	Decision Tree	Random Forest	Tree Augmented Naive Bayes
**Logistic Regression**	<0.0001	<0.01	0.07
**Decision Tree**	-	<0.0001	<0.001
**Random Forest**	-	-	0.92

**Table 7:** Patient statistics in each bicluster.

Bicluster	N	Age	First $O_{2}$ Saturation	% ICU	% Deceased
1	845	62 ± 15 [0–98]	94 ± 4	0.7	**1.5**
2	555	69 ± 16 [26–105]	91 ± 8	2.3	**14.2**
3	223	78 ± 15 [0–106]	89 ± 9	4.5	**63.2**
4	72	68 ± 12 [34–98]	85 ± 13	70.8	**66.7**

## Abbreviations

The following abbreviations are used in this manuscript:

AI	Artificial Intelligence
AIC	Akaike’s Information Criterion
AUC	Area Under the Curve
PRC	Precision Recall Curve
BN	Bayesian Network
ER	Emergency Room
ML	Machine Learning
NPV	Negative Predictive Value
PPV	Positive Predictive Value
RF	Random Forest
ROC	Receiver Operating Curve
TAN	Tree Augmented naive Bayes Network
WHO	World Health Organization
